# RNA-sequencing data highlighting the time-of-day-dependent transcriptome of the central circadian pacemaker in *Sox2*-deficient mice

**DOI:** 10.1016/j.dib.2019.103909

**Published:** 2019-04-08

**Authors:** Arthur H. Cheng, Pascale Bouchard-Cannon, Rob W. Ness, Hai-Ying Mary Cheng

**Affiliations:** a Department of Biology, University of Toronto Mississauga, Mississauga, ON, L5L 1C6, Canada; b Department of Cell & Systems Biology, University of Toronto, Toronto, ON, M5S 3G5, Canada; c Department of Ecology & Evolutionary Biology, University of Toronto, ON, M5S 3B2, Canada

**Keywords:** Transcriptomics, Circadian rhythms, SOX2, Suprachiasmatic nucleus, Mice

## Abstract

SOX2 is a stem cell-associated pluripotency transcription factor whose role in neuronal populations is undefined. Here we present the RNA-sequencing based transcriptome profiles of control (*Sox2*^*fl/fl*^) and SOX2 conditional knock-out (*Vgat-cre;Sox2*^*fl/fl*^) mice at four time points in one 24-h circadian cycle. The raw sequencing data were deposited to ArrayExpress database at EMBL-EBI (https://www.ebi.ac.uk/arrayexpress) under the accession number E-MTAB-7496. Results of rhythmicity analysis, differential expression analysis, network prediction, and potential target identification stemming from the RNA-sequencing dataset are also given in this article. The interpretation and discussion of these data can be found in the related research article entitled “SOX2-dependent transcription in clock neurons promotes the robustness of the central circadian pacemaker.” Cheng et al. 2019.

**Table:** Specifications table

Subject area	Biology
More specific subject area	Circadian rhythm; chronobiology
Type of data	Tables, figures and graphs
How data were acquired	RNA sequencing using Illumina HiSeq4000 platform
Data format	Raw and analyzed
Experimental factors	Organism: *Mus musculus* Strain background: C57BL6 x FVB x 129/SvEv Developmental stage: Adult Tissue: Suprachiasmatic nucleus Genotype: *Sox2*^*fl/fl*^ (control) and *Vgat-cre;Sox2*^*fl/fl*^ mice
Experimental features	Mice were maintained on a fixed 12-h light: 12-h dark schedule and released into constant darkness for two consecutive cycles prior to tissue harvest. RNA from the suprachiasmatic nuclei was extracted and libraries were prepared and sequenced.
Data source location	Department of Biology, University of Toronto Mississauga, Canada
Data accessibility	The RNA-seq data are publicly available at the ArrayExpress database at EMBL-EBI (https://www.ebi.ac.uk/arrayexpress) under accession number E-MTAB-7496
Related research article	A.H. Cheng, P. Bouchard-Cannon, S. Hegazi, C. Lowden, S.W. Fung, C.-K. Chiang, R.W. Ness, H.-Y.M. Cheng, SOX2-dependent transcription in clock neurons promotes the robustness of the central circadian pacemaker, *Cell Rep*, **26**, 2019, 3191–3202, https://doi.org/10.1016/j.celrep.2019.02.068. [1]

**Table:** 

**Value of the data** • The dataset reported here is the first RNA-sequencing data of the murine suprachiasmatic nucleus with samplings throughout a complete 24-h circadian cycle • The rhythmicity analysis of the suprachiasmatic nucleus transcriptomes provides foundation and crucial information for future studies of mammalian circadian rhythms • Differentially expressed gene analysis, network prediction, and regulatory target identification provide insight into the role of Sox2 in circadian rhythm regulation

## 1 Data

To understand the effects of *Sox2* ablation within the central circadian pacemaker in mammals, the suprachiasmatic nucleus (SCN), we sequenced the SCN transcriptomes of *Sox2*^*fl/fl*^ (control) and *Vgat-cre;Sox2*^*fl/fl*^ (SOX2 conditional knock-out) mice at 4 circadian times (CT0, 6, 12, and 18; n = 5 per CT). Raw RNA sequencing data were deposited in the ArrayExpress database at EMBL-EBI (E-MTAB-7496). Alignment metrics can be found in Data S1.

**Fig. 1:**
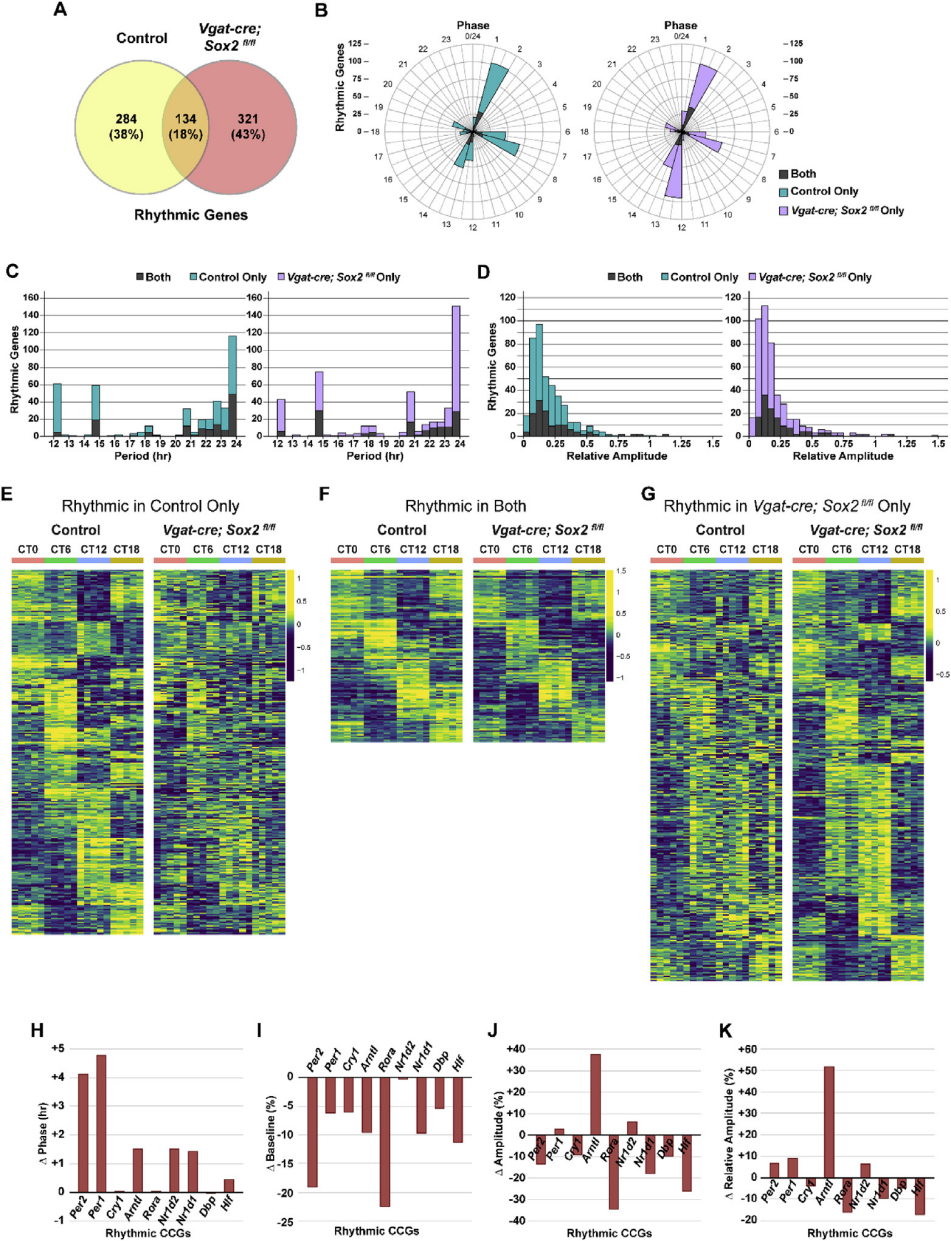
**MetaCycle analysis of control and *Vgat-cre;Sox2***^***fl/fl***^**SCN transcriptomes. (A)** Venn diagram illustrating the overlap between the sets of rhythmic transcripts in the control (yellow) and *Vgat-cre;Sox2*^*fl/fl*^ (pink) SCN. 418 and 455 rhythmic genes were identified in the SCN of control and *Vgat-cre;Sox2*^*fl/fl*^ mice, respectively. **(B)** Rose plots illustrating the phase distribution of rhythmic genes in the control (left) and *Vgat-cre;Sox2*^*fl/fl*^ (right) SCN. The phase distribution of the common, rhythmic genes was similar in both genotypes (Fig. 1B, gray bars). The phase distribution of genotype-specific rhythmic genes was significantly different (Kolmogorov-Smirnov [K–S] test, *p* = 0.009). **(C, D)** Histograms showing the distribution of **(C)** period and **(D)** relative amplitude of rhythmic genes in the control (left) and *Vgat-cre;Sox2*^*fl/fl*^ (right) SCN. There was no significant difference between control and *Vgat-cre;Sox2*^*fl/fl*^ SCN in terms of the period for the entire set of rhythmic genes (Fig. 1C, gray + teal vs. gray + lilac bars). Among the set of common, rhythmic genes, there was a significant reduction in median period in *Vgat-cre;Sox2*^*fl/fl*^ animals (Mann-Whitney [M - W] U test, *p* = 0.011) (Fig. 1C, gray bars). For the genotype-specific rhythmic genes, the median period was significantly longer (M - W U test, *p* = 0.003), and the distribution of period was altered (K-S test, *p* = 0.004), in *Vgat-cre;Sox2*^*fl/fl*^ animals (Fig. 1C, teal vs. lilac bars). The distribution of relative amplitude for the genotype-specific rhythmic genes was different between control and *Sox2*-deficient mice (K-S test, *p* = 0.012) (Fig. 5D, teal vs. lilac bars). **(E**–**G)** Heat map representation of genes that are rhythmic in the SCN of **(E)** control mice only, **(F)** both genotypes, or **(G)***Vgat-cre;Sox2*^*fl/fl*^ mice only, arranged according to phase. Each column represents one biological replicate. **(H**–**K)** Difference in **(H)** phase, **(I)** baseline, **(J)** amplitude, or **(K)** relative amplitude of expression of rhythmic clock genes between *Vgat-cre;Sox2*^*fl/fl*^ mice and controls (knockout value – control value). For (H), positive values indicate a delayed phase, whereas negative values denote an advanced phase in the *Vgat-cre;Sox2*^*fl/fl*^ animals.

Rhythmicity analysis of the SCN transcriptome of control and *Vgat-cre;Sox2*^*fl/fl*^ mice was conducted with MetaCycle and detailed results are summarized in Fig. 1 and Data S2. Phase, period, and relative amplitude of the rhythmic transcriptomes were compiled and shown in Fig. 1B–D. Expression profile of rhythmic genes are visualized by heatmaps in Fig. 1E–G. The changes in rhythmic component of rhythmic core clock genes (CCGs) were calculated and depicted in Fig. 1H–K.

**Fig. 2:**
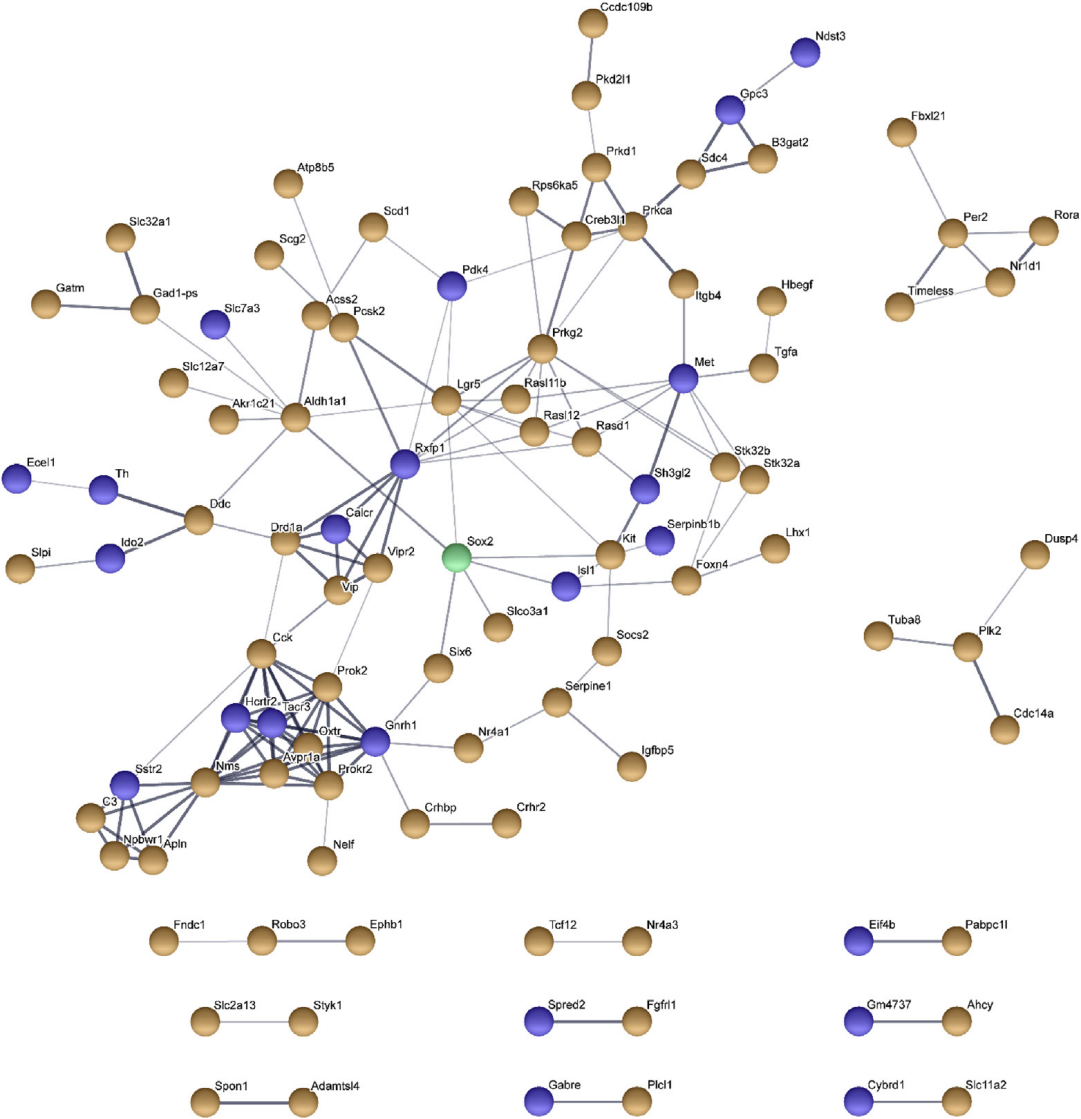
**Protein-protein interaction networks among the set of differentially expressed genes.** 101 DEGs that had at least one predicted interacting partner among the set of 211 mapped DEGs are shown. The largest interaction network is comprised of 73 proteins. Orange denotes DEGs that are downregulated in *Vgat-cre;Sox2*^*fl/fl*^ SCN, and blue indicates upregulated DEGs. SOX2 is shown in green.

**Fig. 3:**
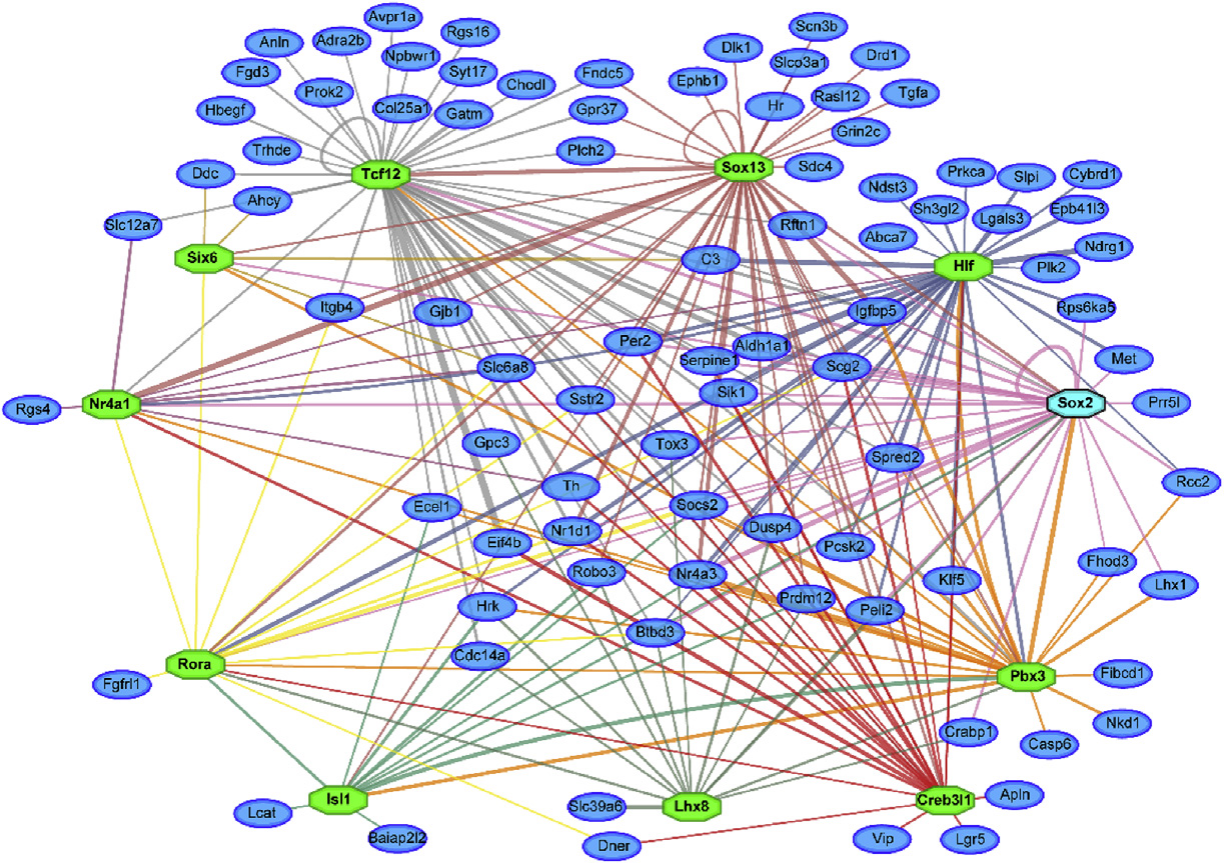
**Amalgamated metatargetome of 11 differentially expressed transcription factors.** Using iRegulon, predicted targets (blue) of transcription factors (green, turquoise) that are differentially expressed in the SCN of *Vgat-cre;Sox2*^*fl/fl*^ mice were identified among the set of DEGs. The network consists of 99 DEGs (out of 233, 42.5%).

**Fig. 4:**
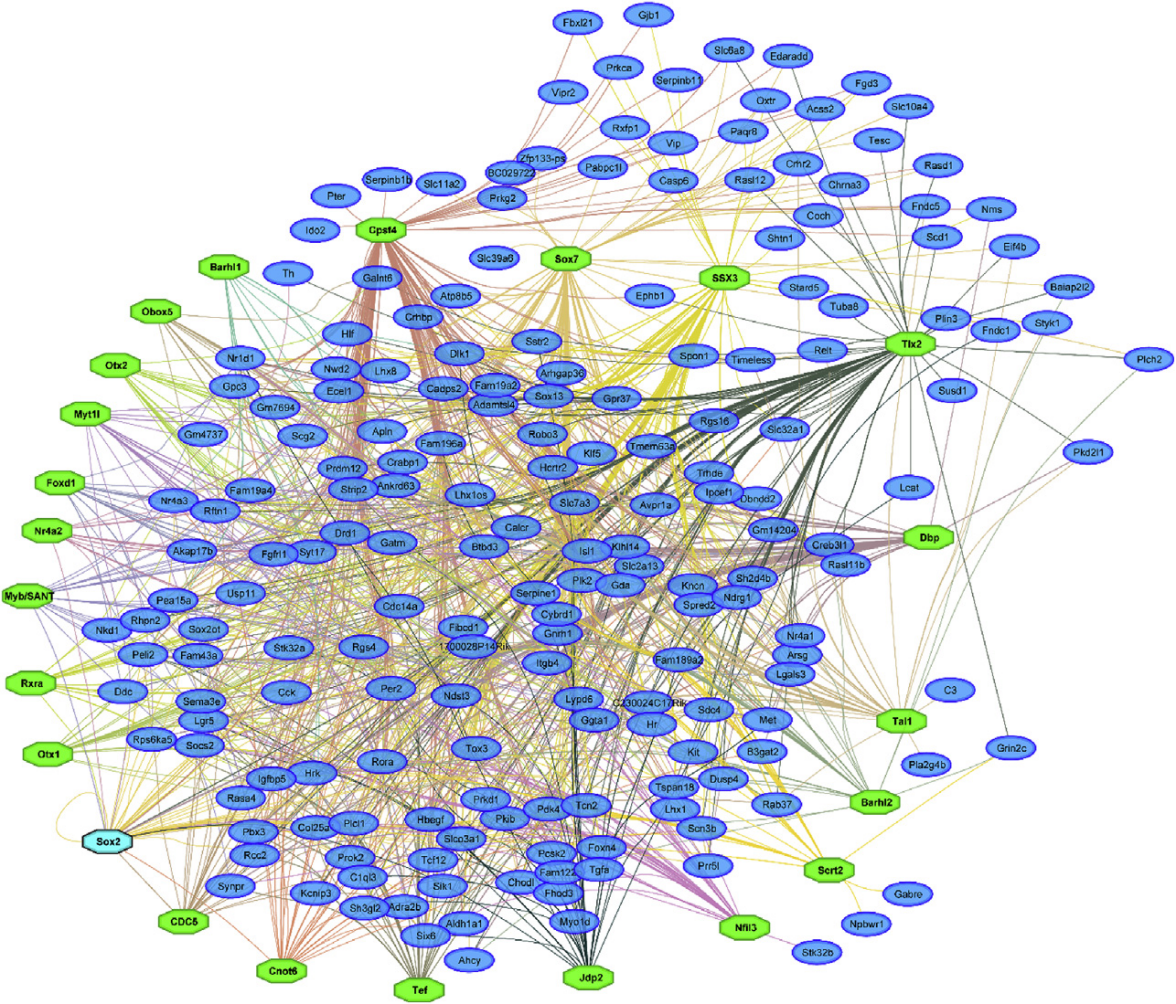
**Transcription factor networks with significantly enriched DNA-binding motifs among the set of differentially expressed genes.** Transcription factors (green) recognizing DNA-binding motifs that are significantly enriched among the set of 213 mapped DEGs (blue) were identified with i-cisTarget. 32 TF regulatory motifs were significantly enriched (Data S5), distributed across 22 TFs.

Differential expression analysis for all transcripts was performed with DESeq2, yielding a list of 233 differentially expressed genes (DEGs) with FDR ≤0.05 (Data S3). Protein-protein interaction networks among the set of 233 DEGs was constructed with STRING and presented in Fig. 2 and Data S4. Potential targets of 11 differentially expressed transcription factors were identified with iRegulon and the resulting amalgamated metatargetome is shown in Fig. 3 and Data S5. Transcription factor networks with enriched regulatory features among the set of 233 DEGs were combined with the metatargetome of SOX2 and presented in Fig. 4 and Data S6.

**Fig. 5:**
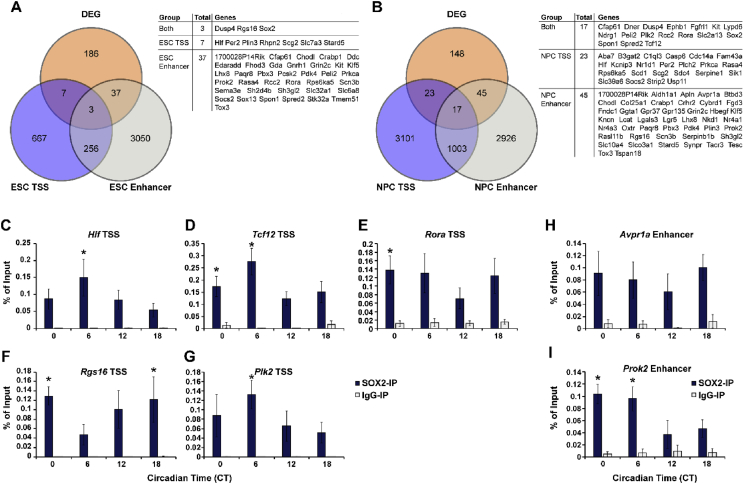
**Identification of potential SOX2 target genes among the set of DEGs and their validation by ChIP-qPCR. (A**–**B)** Venn diagrams illustrating the overlap between our DEG dataset and the SOX2 ChIP-seq datasets obtained from **(A)** ES cells and **(B)** NPCs, as reported in Lodato et al. (2013). The Lodato study distinguishes between SOX2 binding to TSS regions vs. binding to distal enhancer elements of the same gene. The tables list the genes that show SOX2 binding to both TSS and enhancer regions, TSS regions only, or enhancer regions only. **(C**–**I)** ChIP-qPCR analyses of the relative binding of SOX2 to the TSS regions of **(C)***Hlf*, **(D)***Tcf12*, **(E)***Rora*, **(F)***Rgs16*, and **(G)***Plk2*, or to the distal enhancer regions of **(H)***Avpr1a* and **(I)***Prok2* in SCN tissues harvested at 4 CTs. Values are represented as mean ± SEM. n = 3–6 per condition. *p<0.05 vs. IgG-IP.

The list of DEGs was then compared with a previously reported SOX2 ChIP-seq dataset from murine embryonic stem cells (ESCs) and ESC-derived neural progenitor cells (NPC). The gene overlap from this cross-referencing is documented in Fig. 5A–B and Data S7. The binding of SOX2 to 7 predicted genomic regions was validated in SCN tissues by ChIP-qPCR (Fig. 5C–I).

## 2 Experimental design, materials, and methods

### 2.1 Sample collection and RNA sequencing

All animal handling and experimental procedures were performed at the University of Toronto Mississauga (UTM) Animal Facility and were approved by the UTM Animal Care Committee, complying with guidelines established by the University of Toronto Animal Care Committee and the Canadian Council on Animal Care. Detailed procedures for tissue harvesting, RNA extraction, library preparation, high throughput sequencing, read trimming, read mapping, and transcript annotation were described in the related research article [1]. Briefly, SCN tissues from control and *Vgat-cre;Sox2*^*fl/fl*^ mice were harvested at circadian time (CT) 0, 6, 12, and 18. RNA were extracted and then submitted to the Génome Québec Innovation Centre at McGill University (Montréal, Canada) for RNA-seq library preparation. RNA libraries were sequenced on an Illumina HiSeq4000 platform and the reads were trimmed using Trimmomatic v.0.36. Reads for each replicate were aligned to the *Mus musculus* reference genome (GRCm38) using Bowtie2. The *Mus musculus* gene annotation from Ensembl (release 90) was loaded as gene model using makeTxDbFromGFF from the GenomicFeatures package. The function summarizeOverlaps from the GenomicAlignments package was used to generate a SummarizedExperiment object that contained metadata and number of reads per gene. Genes with counts-per-million (cpm) below 0.1 in at least 35 of the 40 libraries were not considered to be expressed in the SCN and were thus discarded from further analysis. Expression data of the remaining 26,798 genes were used for downstream analysis.

### 2.2 Bioinformatics and *in silico* analysis

To identify rhythmic genes, meta2d algorithm from the R package MetaCycle was used on the normalized counts of 26,798 expressed genes in the SCN [3]. Minimum and maximum period were set to 12 and 24 hours, respectively. “JTK” and “LS” integrated period, phase, amplitude, relative amplitude, and benjamini-hochberg q (BH.Q) values were used to compare control and *Vgat-cre;Sox2*^*fl/fl*^ SCN transcriptomes. Genes showing a significant profile (BH.Q < 0.05) were classified as rhythmic genes.

For constructing heatmaps and hierarchical clustering of rhythmic genes, variance stabilizing transformations (VST) were first applied to the normalized counts to remove the dependence of the variance on the mean [4]. The amount by which each gene deviates in a specific sample from the average VST transformed values across all samples was used to construct clustered heat maps.

Differential expression analysis was performed using the DESeq2 package with the assumption of negative binomial distribution for RNA-seq data [2]. For each time point, genes were considered differentially expressed in *Vgat-cre;Sox2*^*fl/fl*^ SCN compared to controls when the reported benjamini-hochberg adjusted p values (PADJ) is less than 0.05.

Protein-protein interaction networks among the set of 233 DEGs was constructed with STRING [5]. Out of 211 mapped DEGs, 101 were predicted to interact with at least one other DEG. There was a total of 163 interactions, with the largest network consisting of 73 DEGs.

Using Cytoscape plugin iRegulon [6], we amalgamated the metatargetome of 11 differentially expressed transcription factors with positive queries. The occurrence count threshold and number of nodes to return were set to 5 and 1000, respectively. GeneSigDB, Ganesh clusters, and MSigDB were used as the databases of signatures/gene sets.

The 233 DEGs were submitted to i-cisTarget regulatory features and cis-regulatory modules prediction [7]. Minimum fraction of overlap, normalized enrichment score (NES) threshold, and AUC threshold were set to 0.4, 3.0, and 0.005, respectively. Enriched predicted regulatory motifs and the associated transcription factor were documented in Data S6. Number of potential regulatory features were calculated for each regulator-target pair and represented by the stroke weight in the regulatory network. DEGs with potential SOX2 binding sites were identified with iRegulon and mapped onto the same network.

ChIP-seq data of bound promoters, active enhancers, and poised enhancers for SOX2 in ESCs and NPCs were extracted from Table S2 of Lodato et al. (2013) [8]. Active enhancers and poised enhancers were grouped together for comparison. The list of SOX2 binding sites were cross-checked with our list of DEGs. Seven genes from the set of DEGs that overlapped with the Lodato dataset were selected and binding of SOX2 to their reported regulatory region was confirmed with ChIP-qPCR using SCN tissues extracted at four CTs. Comprehensive procedure on tissue harvesting, ChIP, and qPCR were described in the related research article [1].

### 2.3 Statistical analysis

Data were analyzed using two-way ANOVA, Mann-Whitney U test, and Kolmogorov-Smirnov test with R version 3.5.0. Post hoc significance of pairwise comparisons was assessed using Tukey's Honest Significant Difference test with α set at 0.05.

## References

[1] Cheng A.H., Bouchard-Cannon P., Hegazi S., Lowden V., Fung S.W., Chiang C.-K., Ness R.W., Cheng H.-Y.M. (2019). SOX2-dependent transcription in clock neurons promotes the robustness of the central circadian pacemaker. Cell Rep..

[2] Love M.I., Huber W., Anders S. (2014). Moderated estimation of fold change and dispersion for RNA-seq data with DESeq2. Genome Biol..

[3] Wu G., Anafi R.C., Hughes M.E., Kornacker K., Hogenesch J.B. (2016). MetaCycle: an integrated R package to evaluate periodicity in large scale data. Bioinformatics.

[4] Anders S., Huber W. (2010). Differential expression analysis for sequence count data. Genome Biol..

[5] Szklarczyk D., Morris J.H., Cook H., Kuhn M., Wyder S., Simonovic M., Santos A., Doncheva N.T., Roth A., Bork P., Jensen L.J., Von Mering C. (2017). The STRING database in 2017: quality-controlled protein-protein association networks, made broadly accessible. Nucleic Acids Res..

[6] Janky R., Verfaillie A., Imrichová H., van de Sande B., Standaert L., Christiaens V., Hulselmans G., Herten K., Naval Sanchez M., Potier D., Svetlichnyy D., Kalender Atak Z., Fiers M., Marine J.C., Aerts S. (2014). iRegulon: from a gene list to a gene regulatory network using large motif and track collections. PLoS Comput. Biol..

[7] Imrichová H., Hulselmans G., Kalender Atak Z., Potier D., Aerts S. (2015). i-cisTarget 2015 update: generalized cis-regulatory enrichment analysis in human, mouse and fly. Nucleic Acids Res..

[8] Lodato M.A., Ng C.W., Wamstad J.A., Cheng A.W., Thai K.K., Fraenkel E., Jaenisch R., Boyer L.A. (2013). SOX2 Co-occupies distal enhancer elements with distinct POU factors in ESCs and NPCs to specify cell state. PLoS Genet..

